# Evidence of Inflammatory System Involvement in Parkinson's Disease

**DOI:** 10.1155/2014/308654

**Published:** 2014-06-24

**Authors:** Yinxia Chao, Siew Cheng Wong, Eng King Tan

**Affiliations:** ^1^National Neuroscience Institute, Singapore 308433; ^2^Duke-National University of Singapore Graduate Medical School, Singapore 169857; ^3^Singapore Immunology Network, Agency for Science, Technology and Research, Singapore 138648; ^4^Department of Microbiology, National University of Singapore, Singapore 117545; ^5^Department of Neurology, Singapore General Hospital, Singapore 169608; ^6^Department of Neurology, National Neuroscience Institute (SGH Campus), 20 College Road, Academia Level 4, Singapore 169856

## Abstract

Parkinson's disease (PD) is a chronic neurodegenerative disease underpinned by both genetic and environmental etiologic factors. Recent findings suggest that inflammation may be a pathogenic factor in the onset and progression of both familial and sporadic PD. Understanding the precise role of inflammatory factors in PD will likely lead to understanding of how the disease arises. *In vivo* evidence for inflammation in PD includes dysregulated molecular mediators such as cytokines, complement system and its receptors, resident microglial activation, peripheral immune cells invasion, and altered composition and phenotype of peripheral immune cells. The growing awareness of these factors has prompted novel approaches to modulate the immune system, although it remains whether these approaches can be used in humans. Influences of ageing and differential exposure to environmental agents suggest potential host-pathogen specific pathophysiologic factors. There is a clear need for research to further unravel the pathophysiologic role of immunity in PD, with the potential of developing new therapeutic targets for this debilitating condition.

## 1. Introduction

**Figure 1:**
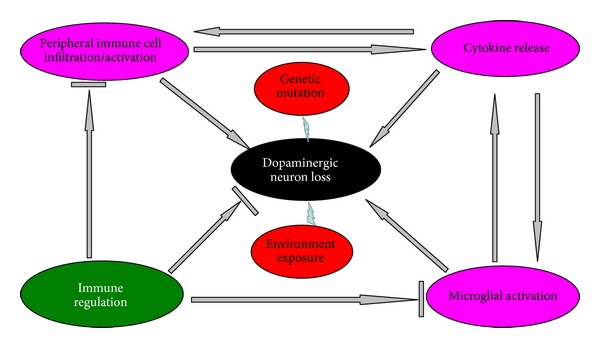
The paper is summarized in the schematic drawing. Genetic mutations and environmental exposures can selectively induce dopaminergic neuron loss in the SNc. The mechanism may involve both regional (microglia) and systematic (peripheral) immune dysregulation. The dysregulated immune system can either elicit or exacerbate dopaminergic neuron loss at SNc by direct contact or by overrelease of cytokines and other immune mediators and thus immune regulation may have great therapeutic potential in PD treatment.

Parkinson's disease (PD), characterized by a loss of dopaminergic neuron, is a common movement disorder, affecting over 4 million individuals worldwide. Although a subject of intense research, the mechanisms underlying PD pathogenesis remain incompletely understood. However, a broad range of studies conducted over the past few decades, including epidemiological, genetic, and postmortem analysis, as well as* in vitro* and* in vivo* modeling, have contributed significantly to our understanding of the pathogenesis of the disease. It is generally accepted that both genetic and environmental factors contribute to the development of PD. Several genes have been identified including* SNCA, PARKIN, DJ-1, PINK1,* and* LRRK2*, whose mutations are responsible for rare familial forms of PD. Despite such progress, the functions of the products of most susceptibility genes have not been fully elucidated. The large number of susceptibility genes probably reflects the complexity of the pathogenesis that contributes to the development of PD. While genetic and chemical models of the disease have established oxidative stress and mitochondrial and proteasomal dysfunction as disease-perpetrating events, the mechanism mediating dopaminergic deficit in sporadic PD remains unknown [1]. The search for a PD pathoetiologic mechanism has uncovered dysfunction of the immune system, particularly innate neuroinflammatory response, as a potential etiologic factor [2, 3]. In addition, recent reports have also implicated the adaptive immune system in PD pathogenesis. Research on susceptibility genes identified by GWAS indicates that some autoimmune diseases such as Crohn's disease may share mutations on the same gene* LRRK2*, which has exemplified the significance of immune system in the pathogenesis of PD. Interestingly, other PD genes have also been reported to have a functional role in immune system. DJ-1 has been reported to regulate mast cell activation and IgE-mediated allergic responses [4]. Manzanillo and colleagues have shown that parkin has a role in ubiquitin-mediated autophagy of* M. tuberculosis*. They reported that both parkin-deficient mice and flies are sensitive to various intracellular bacterial infections [5]. We propose that the likelihood of a common mechanism fundamental to the etiology of all genetic backgrounds of PD is the dysregulation of the immune system, which makes the patients vulnerable to environmental challenge, such as infections or chemical exposure. Although it is still far away to conclude that PD is an autoimmune disease, a better understanding of the cross-talk between the immune system and the CNS will be crucial to harness natural beneficial responses for therapeutic strategies. Here, we review the involvement of immune system (Figure 1) and inflammatory factors (Table 1) in the pathogenesis of PD and discuss potential therapeutic targets of immune regulation in PD.

## 2. Molecular Mediators

### 2.1. Cytokines and Other Soluble Signaling Proteins

Although the etiology of PD is unknown, it is generally believed that the genetic and environmental factors cause the damage of dopaminergic neurons. Necrosis of neurons is observed following acute brain injury and neurotropic infections or as a result of the release of damaging chemicals such as glutamate, nitric oxide (NO), and reactive oxygen species (ROS). Damaged neurons themselves then induce the local inflammation and may exacerbate immune-mediated disease. Alternative explanation indicates that neuroinflammation may serve as an initiation factor of DA neuron degeneration. This has been supported by researchers using ploy(I:C) (TLR3 agonist) and LPS (TLR4 agonist) to induce the loss of DA neuron at the SNc of murine [6, 7]. The expression and function of PD genes such as LRRK2 and SNCA in immune cells are also in support with this possibility [4, 5]. Mutations of these genes may increase the individual's vulnerability to infections and activate the immune system and lead to dopaminergic neuron demise [8, 9].

Abundant evidence in humans demonstrates a role for chronic inflammation and innate immune activation in PD. Cytokines such as IL-2, IL-4, IL-6, IL-10, TNF-*α*, and IFN-*γ* increased in the serum of PD patients [10, 11] and association between systemic markers of inflammation and idiopathic PD risk has been reported [12]. Postmortem studies found upregulated levels of cytokines (including IL-1, TGF-*α*, IFN-*γ*, and IL-6) in the CSF and nigrostriatal regions of individuals with PD relative to age-matched healthy controls [13–16]. Of particular interest, matrix metalloproteinase-3 (MMP-3) can stimulate microglia to produce proinflammatory and cytotoxic molecules such as TNF-*α*, IL-6, and IL-1*β* as well as MMP-3, which in turn contribute to neuronal damage [17]. MMP-3 has also been reported to damage blood-brain barrier (BBB) and amplify neuroinflammation in an MPTP mouse model of Parkinson's disease [18]. In addition, MMP-3 can be induced in astrocyte by polyI:C and impair neurodevelopment [19]. Furthermore, proteins of the complement system, a serum-mediated mechanism designed to clear antibody and various immune targets, are found in extra neuronal Lewy body postmortem. IL-18 synergizes with IL-12 to produce IFN-*γ* in NK cells [20, 21] and IL-12, IFN-*γ*, and TNF-*α* in monocyte [22]. IL-18 and its receptor in multiple sclerosis (MS) have been intensively studied [23–25] while its role in PD is under investigation. In animal models, IL-18 null mice showed reduced dopaminergic neuron loss upon 1-methyl-4-phenyl-1,2,3,6-tetrahydropyridine (MPTP) treatment [26]. Interestingly, IL-18 gene promoter polymorphism [27] and IL-17 and IL-10 gene polymorphism [28] have been reported to be associated with the risk for developing sporadic PD in the Han Chinese populations. This finding suggests that innate immune activation occurred in association with or in response to Lewy body formation [29]. Serum levels of TNF are elevated in PD patients and the serum levels of IL-6 correlate with Hoehn and Yahr staging [30]. Taken together, these observations indicate that an active inflammatory process with definite innate immune involvement is ongoing in the CNS of PD patients.

### 2.2. Toll-Like and Other Pattern Recognition Receptors

Innate immune responses are generally initiated following recognition of pathogen-associated molecular patterns (PAMPS), conserved structures expressed by infectious agents. Equally important are endogenous signals for innate responses, known as damage-associated molecular patterns (DAMPS), which are nuclear and cytosolic proteins including DNA, heat shock proteins (HSP), ATP, oxidized membrane lipids, and aggregated and modified or misfolded proteins. It has been recently shown that extracellular *α*-synuclein may act as a DAMP for microglia, increasing the expression of TLR-1, -2, -3, and -7, MyD88, MMP-9, TNF-*α*, and IL-1*β* [31].

The innate immunity sensors include both cell-associated pattern recognition receptors (PRRs), which include toll-like receptors (TLRs), NOD-like receptors (NLR), RIG-like receptors (RLRs), C-type lectin receptors, scavenger receptors, and N-Formyl met-leu-phe (fMLP) receptors, and soluble PRRs, such as complements, collectins, pentraxins, and natural antibodies. For many of these PPRs, their expression pattern and role in neurodegenerative disorders are still under investigation. TLRs play a significant role in noninfectious diseases such as atherosclerosis, asthma, and inflammatory bowel disease (IBD) [32–34]. TLR activation induces several signalling pathways via the intracellular adaptor protein Myd88 and TIR-domain-containing adapter-inducing interferon-*β* (TRIF) and production of a wide range of immune-regulatory mediators such as cytokines and chemokines. The involvement of TLRs in neurodegenerative diseases is evidenced by pathology studies of human disorders, as well as by data from experimental animal models [35–37]. As is so often the case in the context of immune regulation, TLR-mediated responses can both aggravate inflammation or contribute to its control.

Another group of PRRs, namely, NOD-like receptors (NLRs), initiate the assembly of inflammasomes which activate caspases and, in this way, promote production and secretion of mature IL-1*β* and IL-18. A*β* and prion proteins are potent activators of the (NLR family, pyrin domain-containing) NLRP3 inflammasome [38–40]. NLRP1 and NLRP5 are known to contribute to neuronal death [41]. A*β* induced IL-1*β* release in microglia through NLRP3 [42]. Although data mining indicates an upregulation of NLRP4 and a downregulation of NR2C2, which is associated with NLRP10, in the fibroblast cells from LRRK2 mutated patients [43], the direct contribution of NLRs to the pathogenesis of PD has not been reported. Yet other receptors important in neuronal-glia interactions are the purinergic receptors P1 (adenosine) and P2 (ATP) receptors. Experimental evidence indicates that A2A adenosine receptors (ARs) play a pivotal role in the inhibition of inflammatory processes. Ohta and Sitkovsky reported that the stimulation of A2A receptor signaling suppressed the inflammation and inhibited the tissue damage in mouse models of liver injury and endotoxin-induced septic shock [44]. The inhibitory effect was through regulating IL-18 activity [45–48] and thus may be candidate of therapeutic target for IL-18-initiated diseases, including MS, RA, and asthma. Adenosine receptors can mediate both potentially neuroprotective and neurotoxic effects. Their roles in different neurodegenerative diseases are not elucidated yet. In PD, the expression of both P1 and P2 receptors is altered [49, 50]. In attempts to clarify the functional significance of these changes, much attention has been devoted to the role of the adenosine A2A receptor in PD since antagonists for this receptor improve clinical symptoms and protect against toxin-induced neuronal degeneration [51, 52]. Thus, PRRs play an ambivalent role in neurodegenerative diseases, depending on the mode of activation.

### 2.3. The Complement System

Complements, which serve as soluble PRRs in the activation of innate immune system, were found in extra neuronal Lewy bodies postmortem. This has been proposed to be important for elimination of aggregated proteins in PD by phagocytosis by microglial cells expressing complement receptors [29]. However, the broad and often profoundly unregulated expression of complement components indicates that complement activation may equally contribute to the demise of neurons and axons. In the MPTP model, mannose binding lectin was found to be increased in SNc area earlier than the neuronal loss, which indicates that the complement system activation may contribute to the dopaminergic neuron damage [53]. The fact that neurons and oligodendrocytes express low levels of complement regulatory proteins renders these cells particularly vulnerable to complement-associated death [54].

## 3. Microglia, the Resident Immune Cells

Inflammation via activated glial cells has been reported as an important factor responsible for pathogenesis of PD [55–57]. The presence of activated microglia has long been reported in PD patients, but the mechanism and role of this activation remain controversial [58].

The classic activation protects neurons from injury and maintains homeostasis in the brain. After the resolution of the activation, microglia return to the resting state. In PD patients, persistent activation of microglia has been observed [59]. Activated microglia have also been found in the SN and/or striatum in animal models of PD [53, 60–62]. An accumulation of activated microglia around dopaminergic neurons has been found in three postmortem human brains with MPTP-induced Parkinsonism [63]. Taken together, these data indicate a high association between the microglia activation and the dopaminergic neuron degeneration. The recently discovered genetic association between the HLA regions with late-onset sporadic Parkinson's disease [64] strengthens the possible relevance of antigen presenting cells (APC) in PD. Although the exact causal link between microglia activation and dopaminergic neuron injury in PD remains controversial, several lines of evidence have suggested that persistent microglia activation exerts deleterious effects that result in the loss of dopaminergic neurons.

The first harmful effect of microglia was through the activation of NF*κ*B pathway [65, 66] and consequently increased release of proinflammatory cytokines, such as tumor necrosis factor-*α* (TNF-*α*) and interleukin-1*β* (IL-1*β*) [67–69], while it decreased release of anti-inflammatory cytokines such as IL-4, IL-13, IL-10, and tumour growth factor-*β* (TGF-*β*). However, both proinflammatory and anti-inflammatory cytokines were observed to be elevated in other studies [10, 70], indicating the complexity of microglia activation. Persistent exposure to high concentrations of the proinflammatory cytokines threatened the viability of dopaminergic neurons. For example, chronic expression of TNF-*α* or IL-1*β* in the SN of rats can induce the dopaminergic neuron death [69, 71, 72]. Moreover, the TNF receptor, also a death-signaling receptor, has been found to be widely expressed on dopaminergic neurons in human SN pars compacta (SNpc) [73] and contributes to phenotype that likely contributes to the selective vulnerability of dopaminergic neurons in PD patients. Correspondingly, mice carrying homozygous mutant alleles for both TNF receptors 1 and 2 were protected against MPTP-induced dopaminergic neurotoxicity [74]. Activated microglia can also release chemokines and recruit peripheral immune cells to brain parenchyma and this will be reviewed later in this paper.

The second noxious effect of activated microglia in PD was an increased production of reactive oxygen species, such as NO and superoxide. These reactive species can directly cross the membrane of dopaminergic neurons, overwhelm the endogenous antioxidant systems, and ultimately cause oxidative stress and degeneration of dopaminergic neurons [75].

Thirdly, microglia may also present endocytosed or lysosomal peptides to CD4 T lymphocyte through the expression of MHC class II [76, 77], which can propagate the inflammatory process. In light of these findings, activated microglia certainly appear to be toxic and inexorably exacerbate the death of dopaminergic neurons. However, modest activation of microglia is necessary and beneficial for brain health. With respect to their role in promoting neuronal injury, the focus should also be on plasticity of the microglial response rather than identifying them as a solely negative factor.

However, the pinpoint for the microglia transit from protective into neurotoxic ones is not clear. Microglia were activated acutely when dopaminergic neuron death occurred in the SN of many MPTP- or 6-hydroxydopamine- (6-OHDA-) induced animal models, which usually resemble the end stage of clinical PD. However, PD develops gradually and becomes a chronically acquired state. Thus, a complete profile of microglial activation during the entire course of PD may help us reach a better understanding of progression of the disease. In fact, the plasticity of microglial states is dependent on not only the type of stimulus but also the duration and magnitude of stimulation [78]. A delicate equilibrium of microglial-derived factors might determine the neurotrophic or neurotoxic effect of activated microglia. The modulation of microglial functional states may be a useful tool to intervene in the progression of PD.

## 4. Peripheral Leukocyte Infiltration: Recruited Immune Cells from Blood

Primarily taken as an immune privileged organ, the brain parenchyma infiltration of peripheral leukocyte is tightly regulated at the level of the blood-brain barrier (BBB) [79, 80]. However, the peripheral leukocyte migration and infiltration in the brain do occur in PD.

Since the invasion of T cells in the brain was first reported [58, 81], numerous studies have confirmed the infiltration of immune cells in the brain in both postmortem PD patients and animal models [82, 83]. These brain-invading lymphocytes consisted of a heterogeneous population of both CD8+ and CD4+ cells. A similar profile of peripheral leukocyte infiltration was also present in MPTP mouse models of PD, which further validated the relevance of the model to the human syndrome [82]. While still under investigation, the infiltration of regulatory T cells (Treg) was generally accepted to have protective effect on dopaminergic neurons through suppressing microgliosis either by direct contact or secreting cytokines that attenuate inflammatory responses [84, 85]. Interestingly, both the experimental model and the clinical syndrome exhibit BBB dysfunction [53, 86]. Rentzos and colleagues also report that circulating RANTES (Regulated on Activation, Normal T Cell Expressed and Secreted), which is a C-C beta-chemokine with strong chemoattractant activity for T lymphocytes and monocytes [87], was increased in PD patients, which indicates that the recruitment of T lymphocytes to sites of inflammation in the central nervous system of PD patients may be through the interaction of RANTES and its receptor CCR5.

Besides direct function at the inflammatory site, the peripheral immune cells may also get involved in the pathogenesis of PD systemically. CD4+ T and CD19+ B cells have been reported to be reduced in the peripheral blood of PD patients due to a reduced development/proliferation or because the recruitment to the brain is unclear [88]. Another study by Niwa and colleagues showed an increase of NK cells and a decrease of Th1 cells in the peripheral blood and they were associated with disease severity in patients with PD [89]. A strong upregulation of peripheral monocyte percentage and the CCR2 on its surface has also been reported in PD patients [90]. Interestingly, many of the genes identified to be the cause of familial PD have also been found to be expressed in peripheral immune cells and play important roles. The first identified mutation to cause PD was the SNCA gene, which is expressed in a wide variety of immune cells including T cells, B cells, NK cells, microglia, and monocytes [91–94]. LRRK2 and DJ-1 were also found to be expressed in different populations of human peripheral blood mononuclear cells (PBMCs) and may contribute to autoimmune diseases such as Crohn's disease, leprosy [95, 96], and multiple sclerosis [97]. Parkin was recently found to mediate resistance to intracellular pathogens. The authors managed to show that the parkin-deficient mice and flies are sensitive to many intracellular bacterial infections, which include* M. tuberculosis*, indicating that this gene has a conserved role in metazoan innate defence [5]. Taken together, these results suggest the existence of highly regulated immunopathogenic mechanisms at work in PD that may ultimately be harnessed as therapeutic(s).

## 5. PD and Infectious Disease

Since the Poskanzer and Schwab hypothesis (PSH) that Parkinson's disease was due to influenza infection in the 1950s, the relationship between PD and viral infection has received much attention. Although the original hypothesis has been proven to be fault, the association between pathogen exposure and Parkinson's disease is still being actively pursued. Although infection has not been shown to be associated with PD by some researchers [98], more recently, it was reported that viral infections such as mumps, scarlet fever, influenza, whooping cough, and herpes simplex infections were significantly related to PD development [99]. It was also suggested, but never proven, that intrauterine influenza infection may be related to PD. Alpha-synuclein has also recently been proposed as a prion-like protein that can migrate from affected to unaffected neurons [100, 101]. Further evidence is needed to establish the relationship between PD and pathogen infections.

## 6. Immune Regulation as Therapeutic Targets

Evidence for the involvement of the immune system (both peripheral immune cells and brain resident microglia) in the development and progression of PD has inspired immunotherapeutic approaches to prevent neuronal loss and to aid neuronal growth. In general, researchers have tried to block the effects of microglia-derived inflammatory mediators [102] or modulate the peripheral immune system [76, 103]. Strategies aimed at harnessing the inflammatory response in preclinical models of PD have included use of anti-inflammatory gene therapy approaches: overexpression of a dominant negative TNF molecule to block native TNF signalling has been shown to effectively protect neurons from 6-OHDA-induced cell death even after delayed administration [104, 105]. Another approach has been the use of NSAIDs (nonsteroid anti-inflammatory drugs) as their use seems to reduce the risk of PD development [106–109]. Anti-inflammatory compounds, such as naloxone, minocycline, and dexamethasone, can reduce microglia activation and neuronal damage in different models of nigral degeneration [110–113]. Alternatively, the potential neuroprotective compounds may be hydrogen sulphide-releasing l-DOPA derivatives that reach the brain and reduce the level of IL-6/TNF and NO from microglia [114]. More specific blockade of inflammation has been achieved successfully with inhibitors of COX-2 [115], which has been shown to be increased in PD SN [116].

Peripheral immune system modulation has been mainly designed to prime T cells* in vivo* with different agents and then transfer them to the periphery of animal models with induced dopaminergic neuron death. Another common strategy is to induce Treg (regulatory T cells)/tolerance. Indeed, Treg transfer into MPTP-treated animals attenuated loss of nigral DA neurons [84, 85]. Of particular interest is the stem cell transplant therapy. These neural stem cells (NSCs) and mesenchymal stem cells (MSCs) are no longer believed to primarily replace damaged cells but rather to modulate immune responses and rescue the dopaminergic neurons by secreting a variety of soluble factors [53, 117].

## 7. Conclusions

There is increasing evidence that dysregulated inflammatory responses are implicated in PD. While a direct causal relationship between an infectious agent and PD has yet been unequivocally proven, studies of specific host-pathogen responses that are relevant to select individuals will provide further pathophysiologic clues. Based on current evidence, interventions aimed at either blocking microglia-derived inflammatory mediators or modulating the peripheral immune cells may be potentially useful therapies that are worth exploring. The role of PD genes in modulating the immune system will hopefully unravel pathophysiologic clues that could lead to development of new therapeutic targets.

## Figures and Tables

**Table 1:** Inflammatory factors involved in Parkinson's disease.

Cytokines and other soluble molecules	IL-1, IL-2, IL-4, IL-6, IL-10, TNF-*α*, IFN-*γ*, TGF-*α*, IL-6, MMP-3, IL-17, and IL-18

Pattern recognition receptors (PRRs)	TLRs (TLR-1, -2, -3, and -7), NLRs, and complements

Immune cells	Microglia, monocyte, NK cell, T-cell, and B cell
